# Travel Time to Treating Facility and Mortality in Men With Prostate Cancer

**DOI:** 10.1001/jamanetworkopen.2025.46812

**Published:** 2025-12-03

**Authors:** Stephan M. Korn, Filippo Dagnino, Danesha Daniels, Zhiyu Qian, Hanna Zurl, Klara K. Pohl, Mei-Chin Hsieh, Brenda Y. Hernandez, Andrea Piccolini, Alexander P. Cole, Amanda J. Reich, Joel S. Weissman, Quoc-Dien Trinh, Hari S. Iyer

**Affiliations:** 1 Department of Urology Brigham and Women’s Hospital, Harvard Medical School, Boston, Massachusetts; 2 Center for Surgery and Public Health, Brigham and Women’s Hospital, Harvard Medical School, Boston, Massachusetts; 3 Department of Urology, Medical University of Vienna, Vienna, Austria; 4 Department of Urology, Humanitas Research Hospital–IRCCS, Milan, Italy; 5 Department of Biomedical Sciences, Humanitas University, Milan, Italy; 6 Department of Urology, Medical University of Graz, Graz, Austria; 7 Louisiana Tumor Registry and Epidemiology and Population Health, Louisiana State University Health Sciences Center School of Public Health, New Orleans; 8 The Hawaii Tumor Registry, University of Hawaii Cancer Center, Honolulu; 9 Department of Urology, University of Pittsburgh, Pittsburgh, Pennsylvania; 10 Section of Cancer Epidemiology and Health Outcomes, Rutgers Cancer Institute, New Brunswick, New Jersey

## Abstract

**Importance:**

Geographic access to prostate cancer care may influence outcomes but is often measured using straight-line distance, which may not accurately capture the travel burden experienced by patients.

**Objective:**

To evaluate the association between estimated travel time from patients’ residence to treatment facilities and all-cause and prostate cancer–specific mortality.

**Design, Setting, and Participants:**

This retrospective cohort study used data from the Multilevel Epidemiologic Tumor Registry for Oncology, a nationally representative, investigator-initiated, population-based cancer registry that integrates geomasked residential and treatment facility information from multiple US state cancer registries. The study included patients aged 40 to 99 years from Hawaii, Louisiana, Massachusetts, New Jersey, Ohio, Utah, and Washington (Seattle and Puget Sound areas) with prostate cancer diagnosed between January 1, 2000, and December 31, 2015, and follow-up through January 1, 2018. Patients with missing follow-up, diagnosis at autopsy, and missing residential or treatment facility data were excluded. The data were analyzed from May 1, 2024, to March 15, 2025.

**Exposure:**

Travel time from residence to treatment facility, categorized as less than 30 minutes vs 30 minutes or longer, was estimated based on masked geocodes and road network data.

**Main Outcomes and Measures:**

The primary outcomes were all-cause and prostate cancer–specific mortality measured using Cox proportional hazards regression models.

**Results:**

The study included 159 943 men (mean [SD] age, 66.3 [9.5] years; median [IQR] follow-up, 101.2 [57.3-120.0] months), of whom 44.1% were estimated to have a less than 30-minute travel time and 55.9% to have a 30-minute or longer travel time. Those with a long travel time had a lower risk of death from any cause (adjusted hazard ratio, 0.91 [95% CI, 0.89-0.93]) and from prostate cancer (adjusted hazard ratio, 0.90 [95% CI, 0.86-0.95]).

**Conclusions and Relevance:**

This cohort study found that longer travel time to treatment facilities was associated with lower all-cause and prostate cancer–specific mortality, possibly reflecting better quality through care centralization at specialized centers. While consistent across most subgroups, the findings should be interpreted with consideration of access barriers, such as transportation, that may contribute to missed or delayed diagnoses.

## Key Points

**Question:**

Is longer travel time to treatment associated with survival among patients with prostate cancer?

**Findings:**

In this cohort study of 159 943 patients with prostate cancer from 7 US states, those with travel times of 30 minutes or longer had significantly lower mortality rates compared with those with travel times of less than 30 minutes. There was 9% lower all-cause and 10% lower prostate cancer–specific mortality among patients with longer travel times compared with shorter travel times.

**Meaning:**

These findings suggest that the observed survival advantage with longer travel time may reflect centralization benefits, though ensuring equitable health care access for all patients remains an important consideration.

## Teaser

This cohort study evaluates the association between estimated travel time from patients’ residence to treatment facilities and all-cause and prostate cancer–specific mortality in 7 US states.

## Introduction

Prostate cancer is the second most common cause of cancer-related deaths among men in the US.^1^ Beyond disease-specific factors, sociodemographics, such as race and ethnicity, socioeconomic status, and insurance status, can substantially influence prostate cancer outcomes throughout the disease course.^2,3,4,5,6^

Health care travel is being recognized as an important access barrier for socioeconomically disadvantaged populations and racial and ethnic minority groups^7^ and impacts cancer outcomes.^8,9,10^ Greater travel distance correlates with more aggressive localized prostate cancer at diagnosis,^11^ is an independent estimator for radical treatments,^12^ and is associated with lower receipt of radiation therapy.^13,14^ Notably, associations of travel distance with long-term cancer outcomes can vary in direction. Across several cancer sites, greater travel distance to treatment facilities correlates with improved survival,^8,15^ a pattern also observed for prostate cancer.^16^

Health care travel is often measured by straight-line distance to treatment facilities, which is easier to measure with most geographic databases. However, this measure may not accurately capture the underlying travel burden.^17^ Travel time may represent a more accurate measure. Travel time to seek health care is influenced by patients’ available transportation, socioeconomic resources, and residential location.^17,18^ Furthermore, travel times to health care are considerably longer for Black men than other racial and ethnic groups.^7,19^ This disparity reflects both their greater reliance on public transportation and distances to treatment facilities.^20,21^ Travel time, therefore, may partly explain socioeconomic and racial and ethnic variation in outcomes for prostate cancer, including survival.

Little is known about the association of travel time with long-term prostate cancer outcomes and whether it differs from that of travel distance as noted earlier. Herein, we assessed the role of estimated travel time with prostate cancer–specific and all-cause mortality within a multistate study of US men with prostate cancer, as well as whether travel time explains some portion of racial and ethnic and socioeconomic disparities.

## Methods

### Study Design and Population

This retrospective cohort study analyzed data from the Multilevel Epidemiologic Tumor Registry for Oncology (METRO), a nationally representative, registry-based cohort of patients diagnosed with prostate cancer between January 1, 2000, and December 31, 2015.^22^ The METRO registry aggregates data from multiple state cancer registries that participate in the North American Association of Central Cancer Registries and the Centers for Disease Control and Prevention’s National Program of Cancer Registries, with some also contributing to the National Cancer Institute’s Surveillance, Epidemiology, and End Results (SEER) program. In addition, METRO provides geomasked residential addresses and reporting facility identifiers, allowing analyses at the neighborhood and facility level with greater precision. The institutional review boards of the Dana-Farber Cancer Institute and Rutgers University, The State University of New Jersey, approved this study and determined that because existing data sources were used, no written informed consent was required for participation in the study. The study adhered to the Strengthening the Reporting of Observational Studies in Epidemiology (STROBE) reporting guideline.

### Study Cohort

The study cohort represented patients aged 40 to 99 years newly diagnosed with prostate cancer from 2000 to 2015 from cancer registries in Hawaii, Louisiana, Massachusetts, New Jersey, Ohio, Utah, and Washington. Patient follow-up continued until death, censoring after 10 years, or until January 1, 2018, whichever occurred first. We excluded patients diagnosed with prostate cancer at autopsy and/or had missing follow-up to restrict the cohort to individuals with the potential for treatment. We also excluded patients with missing residential or treatment facility information, both of which are required for travel time calculation (eFigure in Supplement 1).

### Outcomes

Our primary outcomes were all-cause mortality and prostate cancer–specific mortality. Cancer registry data are updated annually through linkage with state death records and the National Death Index, including patients who moved from their original state. For prostate cancer–specific mortality classification, registries use death certificate codes from the *International Classification of Diseases, Ninth Revision* (code 185) and *International Statistical Classification of Diseases, Tenth Revision* (code C61).^23^ For registries contributing to SEER, we used the SEER cause-specific death classification.^23^

### Exposure Variable

Our primary exposure was travel burden, measured as driving time from patient residence to primary treating facility for prostate cancer. Treating facility was assigned using the reporting facility together with the class-of-case field used by registry officials to indicate whether the reporting facility was where diagnosis, treatment, or both occurred. We assumed that the reporting facility was where treatment occurred if there was evidence of definitive treatment (surgery, radiation) or diagnosis otherwise. In settings in which the reporting facility corresponded to a large health system, rather than a single practice or hospital, we assigned the closest facility within that health care system to a patient’s residence. We estimated driving times using a Google Maps–based algorithm implemented via the Travel Time platform in QGIS, version 3.38.0 (Grenoble). We generated isochrone zones (polygons) around each geocoded facility location corresponding to regions with driving times of 15, 30, 45, 60, 75, 90, and 120 minutes around each treating facility at 9:00 am on weekdays between April and June 2024. Patient addresses were geomasked based on the patient’s reporting facility. Travel time isochrones were linked to geomasked patient addresses, allowing us to assign travel times to each patient. This approach provided estimates of patients’ driving times based on street infrastructure and traffic patterns. Driving time was dichotomized as less than 30 minutes vs 30 minutes or longer. A 30-minute travel time has long been proposed as a cutoff for appropriate health care accessibility and was identified as the threshold in which declines in health care service use and access begin to emerge, although its relevance remains debated.^17^ Therefore, we conducted sensitivity analyses using multiple travel time categories.

### Covariates

Sociodemographic covariates in this analysis included age at diagnosis, race and ethnicity (Asian, Native Hawaiian, or Pacific Islander; Hispanic; Non-Hispanic Black; and Non-Hispanic White), insurance status, and year of diagnosis. Neighborhood socioeconomic status (nSES) was calculated following a previously described method using census tract–level data on education, employment, housing, poverty or wealth, racial and ethnic composition, and age, with all variables standardized to generate nSES quintiles 1 (most deprived) to 5 (least deprived).^24^ Race and ethnicity were assigned by cancer registrars based on information recorded in medical records, vital records, and sometimes imputation algorithms based on surname and country of origin. Approximately 5% of our study population were represented by individuals of Asian, Native Hawaiian, and Pacific Islander races and were therefore collapsed into 1 category due to insufficient statistical power to determine reliable associations with outcomes of interest for each separate group. Rural residence was defined as less than 1000 persons per square mile, and urban residence was defined as 1000 persons or more per square mile.^25^ Clinicopathologic covariates included clinical stage and receipt of local therapy.

### Statistical Analysis

We described baseline characteristics stratified by travel time (<30 and ≥30 minutes). Means and SDs were reported for continuous variables and absolute and relative frequencies for categorial variables. Group differences were assessed using Wilcoxon rank sum test for continuous variables and χ^2^ test for categorical variables.

Survival analyses comparing travel times of less than 30 minutes vs 30 minutes or longer included covariates with missing values. Multiple imputation (5 imputed datasets) was used to handle missing data. Covariates included age, race and ethnicity, insurance status (18.9% missing), nSES (0.1% missing), and rural and urban residence (7.3% missing). We used a missing indicator for disease stage (12.9% missing). The imputation model included all aforementioned variables of interest that were subsequently used in the survival analyses.

All-cause mortality and prostate cancer–specific mortality were estimated using the Kaplan-Meier method and compared across travel time groups using the log-rank test. We fitted multivariable Cox proportional hazards regression models to analyze the independent association of travel time groups while adjusting for covariates. Sensitivity analyses were performed using alternative travel time zone groupings (<30, 30 to <60, 60 to <90, and ≥90 minutes) as the exposure. We used cause-specific models for prostate cancer mortality, assuming noninformative censoring for competing deaths conditional on covariates. To assess subgroup differences in both all-cause and prostate cancer–specific mortality, we added interaction terms between travel time (<30 vs ≥30 minutes) and race and ethnicity, age categories, nSES, rural and urban residence, and disease stage (localized, regional, or distant) in separate models. We assessed the proportional hazards assumption using Schoenfeld residual plots^26^ and χ^2^ tests for time trends in residuals for travel time. We found weak evidence of time trends for all-cause mortality but not for prostate cancer–specific mortality, so we included piecewise terms for travel time less than 5 years (early) and 5 years or more (late). We further stratified by state in the all-cause mortality models only because of small event rates and failure of model convergence in prostate cancer mortality models. In each model, results from all imputations were combined, accounting for interimputation and intraimputation variance.^27^

Analyses were conducted from May 1, 2024, to March 15, 2025, using R, version 4.4.1 (R Foundation for Statistical Computing). A 2-sided *P* < .05 was considered significant.

## Results

After exclusions (eFigure in Supplement 1), 159 943 patients newly diagnosed with prostate cancer from 2000 to 2015 (mean [SD] age, 66.3 [9.5] years) were included in our study, of whom 44.1% traveled less than 30 minutes to their treatment facility, while 55.9% traveled 30 minutes or more. Compared with patients with a travel time of less than 30 minutes, those traveling 30 minutes or more were younger (mean [SD] age, 65.9 [9.4] vs 66.7 [9.5] years; *P* < .001). Overall, the distribution of race and ethnicity differed significantly between short (6.8% identified as Asian, Native Hawaiian, or Pacific Islander; 3.5% as Hispanic; 17.6% as Non-Hispanic Black; and 72.1% as Non-Hispanic White race and ethnicity) and long (4.2% identified as Asian, Native Hawaiian, or Pacific Islander; 3.5% as Hispanic; 11.4% as Non-Hispanic Black; and 80.8% as Non-Hispanic White race and ethnicity) travel time groups (*P* < .001). Table 1 provides an overview of the baseline characteristics for the low and high travel burden groups. Compared with excluded participants, those retained in the analytic cohorts were more likely to have private insurance, a missing Gleason score, and missing prostate-specific antigen measures and were more likely to undergo radical prostatectomy (eTable 1 in Supplement 1). eTable 2 in Supplement 1 provides baseline patient characteristics of the final cohort stratified by state, revealing demographic and socioeconomic variation by state. For example, of 39 927 participants in Louisiana, 57.8% resided in the most deprived nSES quintile, whereas of 56 103 participants in Massachusetts, 5.1% resided in the most deprived quintile.

**Table 1.** Descriptive Characteristics of Men With Prostate Cancer at Diagnosis, Stratified by Travel Time

Characteristic	Patients, No. (%)			*P* value
	<30-min Travel time	≥30-min Travel time	Overall	
No. of patients^a^	70 553 (44.1)	89 390 (55.9)	159 943 (100)	NA
Age, mean (SD), y	66.7 (9.5)	65.9 (9.4)	66.3 (9.5)	<.001
Race and ethnicity				
Asian, Native Hawaiian, or Pacific Islander	4766 (6.8)	3747 (4.2)	8513 (5.3)	<.001
Hispanic	2470 (3.5)	3150 (3.5)	5620 (3.5)	
Non-Hispanic Black	12 416 (17.6)	10 226 (11.4)	22 642 (14.2)	
Non-Hispanic White	50 901 (72.1)	72 267 (80.8)	123 168 (77.0)	
nSES, quintile				
1 (Most deprived)	13 169 (18.7)	18 743 (21.0)	31 912 (20.0)	<.001
2	13 052 (18.5)	15 110 (16.9)	28 162 (17.6)	
3	14 240 (20.2)	17 288 (19.3)	31 528 (19.7)	
4	14 870 (21.1)	17 675 (19.8)	32 545 (20.3)	
5 (Least deprived)	13 214 (18.7)	19 001 (21.3)	32 215 (20.1)	
Missing	2008 (2.8)	1573 (1.8)	3581 (2.2)	
Cancer stage				
Localized	49 764 (70.5)	64 953 (72.7)	114 717 (71.7)	<.001
Regional or distant	10 747 (15.2)	13 783 (15.4)	24 530 (15.3)	
Missing	10 042 (14.2)	10 654 (11.9)	20 696 (12.9)	
Insurance status				
Private	29 058 (41.2)	30 410 (34.0)	59 468 (37.2)	<.001
Uninsured	1306 (1.9)	3586 (4.0)	4892 (3.1)	
Medicaid	2299 (3.3)	1687 (1.9)	3986 (2.5)	
Medicare	29643 (42.0)	27 281 (30.5)	56 924 (35.6)	
Other governmental	1894 (2.7)	2512 (2.8)	4406 (2.8)	
Missing	6353 (9.0)	23 914, (26.8)	30 267 (18.9)	
Population density				
Low (<1000 people per square mile)	16 595 (23.5)	41 187 (46.1)	57 782 (36.1)	<.001
High (≥1000 people per square mile)	51 283 (72.7)	46 550 (52.1)	97 833 (61.2)	
Missing	2675 (3.8)	1653 (1.8)	4328 (2.7)	
State				
Hawaii	6117 (8.7)	4296 (4.8)	10 413 (6.5)	<.001
Louisiana	22 923 (32.5)	17 004 (19.0)	39 927 (25.0)	
Massachusetts	29 115 (41.3)	26 988 (30.2)	56 103 (35.1)	
New Jersey	1631 (2.3)	18 786 (21.0)	20 417 (12.8)	
Ohio	4954 (7.0)	1884 (2.1)	6838 (4.3)	
Utah	3458 (4.9)	2344 (2.6)	5802 (3.6)	
Washington (Seattle and Puget Sound areas)	2355 (3.3)	18 088 (20.2)	20 443 (12.8)	
Follow-up, median (IQR), mo	93.4 (50.6-120.0)	107.8 (63.0-120.0)	101.2 (57.3-120.0)	<.001
Definitive treatment (surgery or radiation)	42 969 (60.9)	54 779 (61.3)	97 748 (61.1)	.13

Abbreviations: NA, not applicable; nSES, neighborhood socioeconomic status.

^a^ Percentages by row. All other percentages by column.

Of 159 943 patients overall, all-cause death was recorded for 26.7%, and prostate cancer–specific deaths were recorded for 5.7% over a median follow-up of 101.2 months (IQR, 57.3-120.0 months). The Figure shows the all-cause mortality curves stratified by travel time. In the unadjusted Cox proportional hazards regression model, longer travel time was significantly associated with lower all-cause mortality (hazard ratio [HR], 0.86 [95% CI, 0.84-0.87]). After adjusting for socioeconomic, clinicopathologic, and treatment covariates, patients in the 30 minutes or more travel time group had significantly lower all-cause mortality compared with those in the less than 30 minutes travel time group (adjusted HR [AHR], 0.91 [95% CI, 0.89-0.93]). Table 2 presents HRs for high vs low travel time associated with all-cause and prostate cancer–specific mortality. Higher travel time was associated with a lower risk of prostate cancer–specific death in crude and covariate-adjusted models (HR, 0.82 [95% CI, 0.79-0.86]; AHR, 0.90 [95% CI, 0.86-0.95]). Piecewise analyses showed no major differences in the early vs late period (Table 2). Sensitivity analyses confirmed the robustness of these findings, consistently showing lower mortality among patients with longer travel times compared with those with shorter travel times (eTable 3 in Supplement 1).

**Figure.**
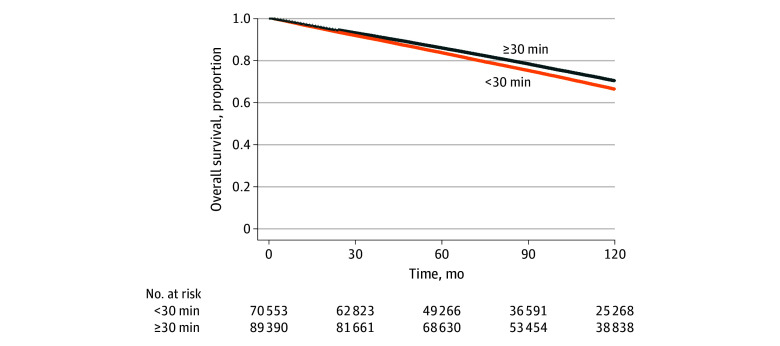
Overall Survival by Travel Time Group Kaplan-Meier survival curves of all-cause mortality by travel burden.

**Table 2.** Association of Long vs Short Travel Time With All-Cause and Prostate Cancer–Specific Mortality Among Men With Prostate Cancer

Model	AHR (95% CI)			
	All-cause mortality		Prostate cancer–specific mortality	
	<30-min Travel time	≥30-min Travel time	<30-min Travel time	≥30-min Travel time
Deaths/person-mo	19 485/5 903 035	22 960/8 043 991	4349/5 903 035	4897/8 043 991
Crude^a^	1 [Reference]	0.86 (0.84-0.87)	1 [Reference]	0.82 (0.79-0.86)
Minimal^b^	1 [Reference]	0.92 (0.90-0.94)	1 [Reference]	0.88 (0.84-0.92)
Full^c^	1 [Reference]	0.91 (0.89-0.93)	1 [Reference]	0.90 (0.86-0.95)
Piecewise				
Early (<5 y)	1 [Reference]	0.93 (0.90-0.95)	NA	NA
Late (≥5 y)	1 [Reference]	0.89 (0.86-0.91)	NA	NA

Abbreviations: AHR, adjusted hazard ratio; NA, not applicable; nSES, neighborhood socioeconomic status.

^a^ Unadjusted.

^b^ Adjusted for age, diagnosis year, race and ethnicity, nSES, population density, and insurance status.

^c^ Minimal model additionally adjusted for stage and receipt of surgery or radiation.

In stratified analyses, the association of longer travel time with lower all-cause mortality was consistent across subgroups (Table 3). The heterogeneity test for race and ethnicity (*P* for heterogeneity = .09) and stage (*P* for heterogeneity = .26) was not statistically significant, suggesting no differences in the overall association between subgroups. The observed association diminished in the least-deprived nSES quintile (AHR, 0.96 [95% CI, 0.91-1.01]; *P* for heterogeneity = .03). Significant heterogeneity emerged for urbanicity (*P* for heterogeneity = .003), with both urban (AHR, 0.87 [95% CI, 0.84-0.90]) and rural (AHR, 0.93 [95% CI, 0.91-0.95]) residence associated with lower all-cause mortality for longer travel time. In state-stratified analyses (*P* for heterogeneity < .001), patients in Massachusetts had the lowest risk of mortality associated with long travel time (AHR, 0.83 [95% CI, 0.80-0.86]) and those living in the Seattle and Puget Sound areas of Washington had the highest risk (AHR, 0.96 [95% CI, 0.89-1.04]).

**Table 3.** Association of Long vs Short Travel Time With All-Cause and Prostate Cancer–Specific Mortality Among Men With Prostate Cancer, Stratified by Race and Ethnicity, nSES, Urbanicity, and Cancer Stage

Variable	AHR (95% CI)^a^	
	All-cause mortality, ≥30-min travel time^b^	Prostate cancer–specific mortality, ≥30-min travel time^b^
Race and ethnicity		
Asian, Native Hawaiian, or Pacific Islander	0.97 (0.89-1.50)	4.07 (2.76-6.00)
Hispanic	1.01 (0.90-1.13)	1.05 (0.84-1.32)
Non-Hispanic Black	0.90 (0.86-0.94)	0.85 (0.77-0.93)
Non-Hispanic White	0.90 (0.88-0.92)	0.87 (0.82-0.91)
*P* for heterogeneity	.09	<.001
nSES, quintile		
1 (Most deprived)	0.87 (0.84-0.91)	0.76 (0.70-0.82)
2	0.90 (0.87-0.94)	0.95 (0.86-1.04)
3	0.89 (0.86-0.93)	0.91 (0.83-1.00)
4	0.94 (0.90-0.98)	0.97 (0.88-1.07)
5 (Least deprived)	0.96 (0.91-1.01)	0.93 (0.84-1.03)
*P* for heterogeneity	.03	<.001
Urbanicity		
<1000 People per square mile	0.87 (0.84-0.90)	0.78 (0.73-0.84)
≥1000 People per square mile	0.93 (0.91-0.95)	0.95 (0.90-1.00)
*P* for heterogeneity	.003	<.001
Cancer stage		
Localized	0.92 (0.90-0.94)	0.91 (0.85-0.98)
Regional or distant	0.90 (0.86-0.94)	0.95 (0.90-1.01)
Missing	0.88 (0.84-0.92)	0.70 (0.63-0.77)
*P* for heterogeneity	.26	<.001
State^c^		
Hawaii	0.96 (0.89-1.03)	NA
Louisiana	0.93 (0.90-0.97)	NA
Massachusetts	0.83 (0.80-0.86)	NA
New Jersey	0.93 (0.85-1.02)	NA
Ohio	0.91 (0.81-1.04)	NA
Utah	0.90 (0.80-1.00)	NA
Washington (Seattle and Puget Sound areas)	0.96 (0.89-1.04)	NA
*P* for heterogeneity	<.001	NA

Abbreviations: AHR, adjusted hazard ratio; NA, not applicable; nSES, neighborhood socioeconomic status.

^a^ Adjusted for age, diagnosis year, race and ethnicity, nSES, population density, insurance status, stage, receipt of surgery or radiation.

^b^ Reference is less than 30-minute travel time.

^c^ Results for prostate cancer–specific mortality not provided due inadequate event rates and failure of model convergence.

For prostate cancer–specific mortality, these patterns were similar, except for race and ethnicity (*P* for heterogeneity < .001), with Asian, Native Hawaiian, or Pacific Islander individuals having a higher risk with longer travel time (AHR, 4.07 [95% CI, 2.76-6.00]). The association of longer travel time and lower prostate cancer mortality diminished across nSES quintiles (*P* for heterogeneity < .001) and cancer stages (*P* for heterogeneity < .001), with significantly higher risks in the most deprived nSES quintile (AHR, 0.76 [95% CI, 0.70-0.82]) and nonsignificant risk among patients with regional or distant disease (AHR, 0.95 [95% CI, 0.90-1.01]).

## Discussion

This multistate cohort study of patients with prostate cancer found that longer travel times were associated with lower all-cause and prostate cancer–specific mortality based on a novel methodology using a geographic routing algorithm to estimate travel time. Furthermore, we found that longer travel time was associated with lower all-cause mortality in most subgroups. These same patterns were observed for prostate cancer–specific mortality for patients of all races and ethnicities except Asian, Native Hawaiian, and Pacific Islander. Together, our findings suggest that centralization of prostate cancer care in specialized centers may provide survival benefits that outweigh the potential barriers of increased travel time.

Our study offers new perspectives in health care access research by focusing on travel time rather than travel distance. Although travel is often a necessary part of accessing health care, the threshold at which travel becomes a barrier remains poorly defined, with up to 10-fold variation reported.^17^ Furthermore, travel is often captured by straight-line distances, whereas travel time may better capture patients’ travel burden.^17,28,29^ We applied a geographic routing algorithm to capture estimated travel times and examine their association with prostate cancer outcomes, which was achieved using the METRO database, an investigator-initiated multistate cohort of men with prostate cancer using National Program of Cancer Registries and SEER registry data. Registry data are linked to neighborhood environmental, health care access, and socioeconomic factors at patients’ residential addresses, creating the basis for analyzing the association between travel burden and prostate cancer outcomes.

It is plausible that the lower mortality associated with longer travel times may represent both centralization and regionalization effects, though our data lack specific hospital-level characteristics to directly evaluate this association. Improved outcomes in multiple cancers, including prostate cancer, have led to centralization of care to high-volume centers.^30,31^ With regionalization, regional networks enable referrals to nearby specialized hospitals. Both centralization and regionalization increase patients’ travel distances, as potentially reflected in our findings.^32,33,34^ Notably, patients in the 30 minutes or longer group were significantly younger (65.9 vs 66.7 years), which may reflect selection of specialized hospitals by individuals who are more health aware. Evidence from England showed that while selection of hospitals that were not the nearest generally declined with longer travel times, younger, healthier, and more affluent men with prostate cancer were more likely to select centers offering robotic surgery or with strong reputations.^35^ Nevertheless, our (age-adjusted) findings also align with previous literature showing a 2% mortality risk per 10-mile increase in travel distance to treatment facilities for patients with prostate cancer.^16^ A French study found that longer travel was generally associated with worse overall survival for various cancer types except prostate cancer.^36^ Travel time in our study was estimated using geomasked rather than exact patient and facility locations, which may have led to nondifferential misclassification of travel burden, particularly around the 30-minute cutoff, potentially biasing the association of longer travel time with mortality. While this threshold followed previous literature,^17^ we conducted sensitivity analyses given the limited evidence supporting this specific cutoff and potential bias from grouping longer travel times. These analyses consistently showed lower mortality rates for patients with travel times of 30 minutes or longer, although they did not account for potential misclassification within this reference group. We found that longer travel time was associated with lower all-cause and prostate cancer–specific mortality consistently across most subgroups. Thus, long-term outcome disparities might be mitigated for individuals receiving care. However, an important exception was observed among Asian, Native Hawaiian, and Pacific Islander patients, for whom longer travel time was associated with a fourfold increased risk of prostate cancer mortality. These populations are heterogeneous, with lower overall prostate cancer incidence and evidence of more aggressive disease at diagnosis and distinct treatment patterns.^37,38^ This finding may reflect both the heterogeneity within Asian, Native Hawaiian, and Pacific Islander populations and differences in state distribution, particularly Hawaii. Given the limited subgroup sizes, however, this finding should be regarded as exploratory and requiring further evaluation, noting that detailed state-level analyses were only feasible for all-cause, but not prostate cancer–specific mortality.

It is important to acknowledge that our study was limited to individuals diagnosed with prostate cancer, reflecting outcomes only among those who accessed health care services. A survey analysis showed that rural patients with a driver’s license or access to a car were twice as likely to attend appointments for chronic condition care, including cancer.^39^ Our study could not determine how travel barriers were associated with individuals seeking care. Substantial travel requirements may lead to missed appointments or avoidance of care^40,41^ or prevent access to screening, diagnosis, and appropriate treatment, potentially resulting in worse survival outcomes.^40^ Further research is needed to explore travel burden and transportation needs across the broader population to ensure equitable health care access and outcomes.

### Limitations

This study had several limitations. Although based on a comprehensive multistate sample, this analysis may not fully represent the US population with prostate cancer but, rather, the included states and their health care (travel) infrastructures. Our analysis focused on initial treating facilities, aligning with previous research protocols.^15,36^ Patients, particularly those with metastatic disease, may have received subsequent care at different institutions. The state registries provided the reporting facility identifier, not necessarily where treatment occurred, which may have introduced measurement error. However, reporting facility codes may offer a more accurate reflection of care patterns than the nearest facility. Using 2024 travel data for a cohort diagnosed between 2000 and 2015 may introduce temporal bias, although longitudinal evidence has indicated only limited changes in median travel times relative to the defined travel time zones.^41^ Assuming nondifferential misclassification of 85% sensitivity and specificity in assigning less than 30 minutes travel time,^42^ we would expect a corrected HR of 0.81 (for all-cause mortality) and 0.76 (for prostate cancer–specific mortality), leaving our interpretation unchanged. Our analysis assumed that health care–related travel occurred via private transportation. However, in a survey of more than 15 000 respondents, 94% used private vehicles for health care trips.^7^ Additionally, we could not account for several survival-associated risk factors, including comorbidities, smoking status, and obesity, or for structural and contextual confounders, such as patient preferences, the availability of specialists, or facility characteristics. These unmeasured factors may have influenced treatment access and outcomes, limiting attribution of observed benefits to centralization.

## Conclusions

This cohort study of patients with prostate cancer diagnosed between 2000 and 2015 with extended follow-up across 7 states found that higher travel burden was associated with lower all-cause and prostate cancer–specific mortality. These findings possibly reflect the beneficial effects of regionalization and centralization of prostate cancer care. Future research should focus on involving individuals who are not able to seek cancer care due to health-related social needs, such as transportation.

## Notes

Supplement 1. **eFigure.** Flowchart of the Study Cohort Selection Process **eTable 1.** Comparison of Participant Characteristics Between Retained and Excluded Groups **eTable 2.** Patient Baseline Characteristics Stratified by State **eTable 3.** Hazard Ratios for Low vs Varying High Travel Time Groups With All-Cause and Prostate Cancer−Specific Mortality Among Men With Prostate Cancer

Supplement 2.
Data Sharing Statement
