# Supplementary material for: Travel Time to Treating Facility and Mortality in Men With Prostate Cancer
Source: JAMA Netw Open. 2025 Dec 3;8(12):e2546812. doi: 10.1001/jamanetworkopen.2025.46812 (PMC12676350; doi:10.1001/jamanetworkopen.2025.46812)
Supplement: Supplement 1. — eFigure. Flowchart of the Study Cohort Selection Process eTable 1. Comparison of Participant Characteristics Between Retained and Excluded Groups eTable 2. Patient Baseline Characteristics Stratified by State eTable 3. Hazard Ratios for Low vs Varying High Travel Time Groups With All-Cause and Prostate Cancer−Specific Mortality Among Men With Prostate Cancer [file jamanetwopen-e2546812-s001.pdf]

Supplemental Online Content

Korn SM, Dagnino F, Daniels D, et al. Travel time to treating facility and mortality in men with prostate cancer. *JAMA Netw Open*. 2025;8(11):e2546812.
doi:10.1001/jamanetworkopen.2025.46812

eFigure. Flowchart of the Study Cohort Selection Process

eTable 1. Comparison of Participant Characteristics Between Retained and Excluded Groups

eTable 2. Patient Baseline Characteristics Stratified by State

eTable 3. Hazard Ratios for Low vs Varying High Travel Time Groups With All-Cause and Prostate Cancer–Specific Mortality Among Men With Prostate Cancer

This supplemental material has been provided by the authors to give readers additional information about their work.

© 2025 Korn SM et al. *JAMA Network Open*.

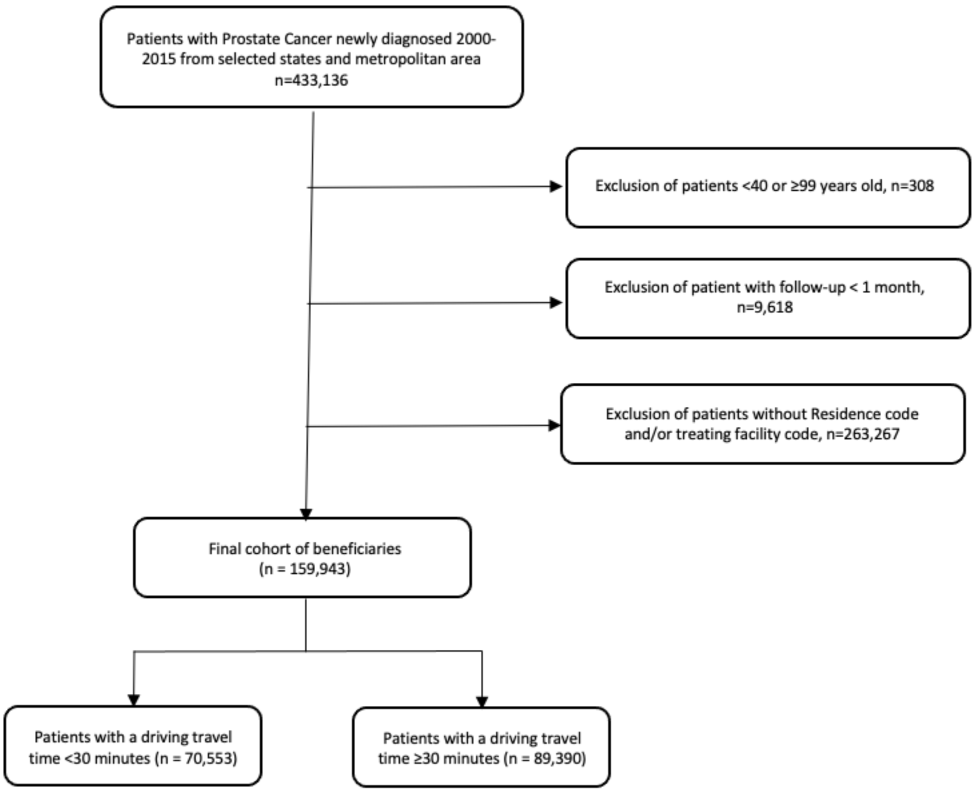

eFigure. Flowchart of the Study Cohort Selection Process

© 2025 Korn SM et al. *JAMA Network Open*.

eTable 1. Comparison of Participant Characteristics Between Retained and Excluded Groups

Characteristic, N (%)	Excluded	Included in Analytic Sample	*P*
N	263 267	159 943	
Age, years (mean (SD))	67.33 (9.56)	66.25 (9.47)	<0.001
Race and ethnicity			<0.001
Asian American, Native Hawaiians and Pacific Islander	9567 (3.6)	8513 (5.3)	
Hispanic	9007 (3.4)	5620 (3.5)	
Non-Hispanic Black	33 259 (12.6)	22 642 (14.2)	
Non-Hispanic White	211 434 (80.3)	123 168 (77.0)	
Year of diagnosis			<0.001
2000-2004	80 511 (30.6)	57 430 (35.9)	
2005-2009	95 852 (36.4)	47 783 (29.9)	
2010-2015	86 802 (33.0)	54 730 (34.2)	
Marital status			<0.001
Not married	47 540 (19.5)	23 382 (22.5)	
Married	158 936 (65.1)	72 592 (70.0)	
Missing	37 683 (15.4)	7784 (7.5)	
Insurance			<0.001
Private	85 332 (32.4)	59 468 (37.2)	
Uninsured	11 856 (4.5)	4892 (3.1)	
Medicaid	3834 (1.5)	3986 (2.5)	
Medicare	105 675 (40.1)	56 924 (35.6)	
Other Government	3246 (1.2)	4406 (2.8)	
Missing	53 324 (20.3)	30 267 (18.9)	
Gleason score			<0.001
<7	84 970 (32.3)	32 311 (20.2)	
7	106 527 (40.5)	37 953 (23.7)	
≥7	39 664 (15.1)	18 386 (11.5)	
Missing	32 106 (12.2)	71 293 (44.6)	
Stage			<0.001
Localized	134 724 (51.2)	114 717 (71.7)	
Regional	15 320 (5.8)	18 222 (11.4)	
Distant	6408 (2.4)	6308 (3.9)	
Missing	106 815 (40.6)	20 696 (12.9)	
Prostate-specific antigen, ng/L			<0.001
<10	108 057 (41.1)	41 415 (25.9)	
10-<20	19 775 (7.5)	7784 (4.9)	
≥20	16 413 (6.2)	5512 (3.4)	
Missing	118 920 (45.2)	105 232 (65.8)	
Surgery			<0.001
No surgery	169 623 (64.4)	85 649 (53.5)	
Localized	2563 (1.0)	1301 (0.8)	
TURP	8364 (3.2)	8001 (5.0)	
Radical prostatectomy	71 493 (27.2)	62 447 (39.0)	
Missing	11 224 (4.3)	2545 (1.6)	
Hormone therapy			<0.001
No	150 424 (57.1)	69 381 (43.4)	

© 2025 Korn SM et al. *JAMA Network Open*.

Yes	55 187 (21.0)	25 757 (16.1)	
Missing	57 656 (21.9)	64 805 (40.5)	
Radiotherapy			<0.001
No	155 110 (58.9)	99 653 (62.3)	
Yes	58 152 (22.1)	38 707 (24.2)	
Missing	50 005 (19.0)	21 583 (13.5)	
Follow-up (median [IQR])	100.70 [60.03, 120.00]	101.23 [57.30, 120.00]	<0.001
nSES (%)			
Quintile 1 (most deprived)	50 498 (19.2)	31 912 (20.0)	
Quintile 2	54 236 (20.6)	28 162 (17.6)	
Quintile 3	50 891 (19.3)	31 528 (19.7)	
Quintile 4	49 853 (18.9)	32 545 (20.3)	
Quintile 5 (least deprived)	50 151 (19.0)	32 215 (20.1)	
Missing	7638 (2.9)	3581 (2.2)	
Population density			<0.001
Low (<1000 people/mi^2)	86 787 (33.0)	57 782 (36.1)	
High (≥1000 people/mi^2)	166 161 (63.1)	97 833 (61.2)	
Missing	10 319 (3.9)	4328 (2.7)	
State			<0.001
Hawaii	2048 (0.8)	10 413 (6.5)	
Louisiana	14 322 (5.4)	39 927 (25.0)	
Massachusetts	14 436 (5.5)	56 103 (35.1)	
New Jersey	81 259 (30.9)	20 417 (12.8)	
Ohio	104 650 (39.8)	6838 (4.3)	
Utah	16 582 (6.3)	5802 (3.6)	
Seattle, Puget Sound (Washington)	29 868 (11.3)	20 443 (12.8)	

Abbreviations: nSES = neighborhood socioeconomic status; SD = Standard Deviation

© 2025 Korn SM et al. *JAMA Network Open*.

eTable 2. Patient Baseline Characteristics Stratified by State								
	States							
**Characteristic, N (%)**	**Hawaii**	**Louisiana**	**Massachusetts**	**New Jersey**	**Ohio**	**Utah**	**Seattle/Puget Sound, Washington**	**Overall**
**N**	**10 413 (6.5)**	**39 927 (25.0)**	**56 103 (35.1)**	**20 417 (12.8)**	**6838 (4.3)**	**5802 (3.6)**	**20 443 (12.8)**	**159 943 (100%)**
Age, years (mean (SD))	68.2 (9.6)	66.1 (9.2)	65.6 (9.4)	66.4 (9.7)	65.3 (8.7)	65.2 (8.9)	68.0 (9.8)	66.3 (9.5)
Race and ethnicity								
Asian American, Native Hawaiians And Pacific Islander	6 561 (63%)	249 (0.6%)	<16[a]	728 (3.6%)	79 (1.2%)	84 (1.4%)	812 (4%)	8 513 (5.3%)
Hispanic	387 (3.7%)	699 (1.8%)	1 857 (3.3%)	2 075 (10.2%)	49 (0.7%)	233 (4%)	320 (1.6%)	5 620 (3.5%)
Non-Hispanic Black	268 (2.6%)	13 379 (33.5%)	3 907 (7%)	3 045 (14.9%)	1 097 (16%)	38 (0.7%)	908 (4.4%)	22 642 (14.2%)
Non-Hispanic White	3 197 (30.7%)	25 600 (64.1%)	50 339 (89.7%)	14 569 (71.4%)	5 613 (82.1%)	5 447 (93.9%)	18 403 (90%)	123 168 (77.0%)
Neighborhood socioeconomic status								
Quintile 1 (Most deprived)	890 (8.5%)	23 072 (57.8%)	2 870 (5.1%)	1 149 (5.6%)	1 575 (23.0%)	518 (8.9%)	1838 (9.0%)	31 912 (20.0%)
Quintile 2	2 016 (19.4%)	8 673 (21.7%)	7 037 (12.5%)	1 997 (9.8%)	2 078 (30.4%)	1 609 (27.7%)	4 752 (23.2%)	28 162 (17.6%)
Quintile 3	1 882 (18.1%)	4 618 (11.6%)	12 306 (21.9%)	3 708 (18.2%)	1 413 (20.7%)	1 556 (26.8%)	6 045 (29.6%)	31 528 (19.7%)
Quintile 4	1 750 (16.8%)	2 491 (6.2%)	16 159 (28.8%)	5 360 (26.3%)	1 052 (15.4%)	1 452 (25.0%)	4 281 (20.9%)	32 545 (20.3%)
Quintile 5 (Least deprived)	373 (3.6%)	1 069 (2.7%)	17 683 (31.5%)	8 180 (40.1%)	716 (10.5%)	667 (11.5%)	3 527 (17.3%)	32 215 (20.1%)
Missing	3 502 (33.65)	<16[a]	48 (0.1)	23 (0.1%)	<16[a]	<16[a]	<16[a]	3581 (2.2%)
Stage								
Localized	8 358 (80.3%)	24 512 (61.4%)	46 203 (82.4%)	13 109 (64.2%)	3 229 (47.2%)	3 022 (52.1%)	16 284 (79.7%)	114 717 (71.7%)
Regional/Distant	1 691 (16.2%)	5 457 (13.7%)	9 229 (16.5%)	2 204 (10.8%)	913 (13.4%)	1 147 (19.8%)	3 889 (19%)	24 530 (15.3%)
Missing	364 (3.5%)	9 958 (24.9%)	671 (1.2%)	5 104 (25%)	2 696 (39.4%)	1 633 (28.1%)	270 (1.3%)	20 696 (12.9%)
Insurance								
Private	3 109 (29.9%)	14 997 (37.6%)	29 586 (52.7%)	5 600 (27.4%)	3 009 (44.0%)	2 663 (45.9%)	504 (2.5%)	59 468 (37.2%)
Uninsured	30 (0.3%)	1 253 (3.1%)	434 (0.8%)	2 929 (14.3%)	121 (1.8%)	122 (2.1%)	<16[a]	4 892 (3.1%)
Medicaid	204 (2.0%)	1 019 (2.6%)	2 072 (3.7%)	358 (1.8%)	255 (3.7%)	64 (1.1%)	<16[a]	3 986 (2.5%)
Medicare	3 575 (34.3%)	17 557 (44.0%)	22 940 (40.9%)	6 485 (31.8%)	3 109 (45.5%)	2 431 (41.9%)	827 (4.0%)	56 924 (35.6%)
Other Govt	1 037 (10.0%)	2 819 (7.1%)	169 (0.3%)	84 (0.4%)	226 (3.3%)	71 (1.2%)	<16[a]	4 406 (2.8%)

© 2025 Korn SM et al. *JAMA Network Open*.

Missing	2 458 (23.6%)	2 282 (5.7%)	902 (1.6%)	4 961 (24.3%)	118 (1.7%)	451 (7.8%)	19095 (93.4%)	30 267 (18.9%)
Population Density								
Low (<1000 people/mi^2)	1 895 (18.2%)	21 188 (53.1%)	19 638 (35.0%)	4 157 (20.4%)	2 671 (39.1)	1 405 (24.2)	6 828 (33.4)	57 782 (36.1%)
High (≥1000 people/mi^2)	5 379 (51.7%)	18 737 (46.9%)	36 241 (64.6%)	15 975 (78.2%)	4 165 (60.9%)	3 723 (64.2%)	13 613 (66.6%)	97 833 (61.2%)
Missing	3 139 (30.1%)	<16[a]	224 (0.4%)	285 (1.4%)	<16[a]	674 (11.6%)	<16[a]	4 328 (2.7%%)
Definitive treatment (surgery or radiation)	6 000 (57.6%)	24 499 (61.4%)	38 355 (68.4%)	11 270 (55.2%)	3 815 (55.8%)	4 064 (70.0%)	9 745 (47.7%)	97 748 (61.1%)

Abbreviations: SD = standard deviation, mi^2 = square mile; [a]Cell counts <16 observations suppressed per registry data regulations

© 2025 Korn SM et al. *JAMA Network Open*.

eTable 3. Hazard Ratios for Low vs Varying High Travel Time Groups With All-Cause and Prostate Cancer–Specific Mortality Among Men With Prostate Cancer

	**All-cause mortality**				**Prostate cancer-specific mortality**			
	**<30 minutes**	**30-<60 minutes**	**60-<90 minutes**	**≥90 minutes**	**<30 minutes**	**30-<60 minutes**	**60-<90 minutes**	**≥90 minutes**
Model	**aHR (95% CI)**	**aHR (95% CI)**	**aHR (95% CI)**	**aHR (95% CI)**	**aHR (95% CI)**	**aHR (95% CI)**	**aHR (95% CI)**	**aHR (95% CI)**
Deaths/person-months	19 485/ 5 903 035	10 453/ 3 778 483	4895/ 1 749 298	7612/ 2 516 210	4349/ 5 903 035	2159/ 3 778 483	1073/ 1 749 298	1665/ 2 516 210
Crude[a]	Ref	0.83 (0.81, 0.85)	0.84 (0.81, 0.87)	0.91 (0.88, 0.93)	Ref	0.76 (0.72, 0.80)	0.85 (0.79, 0.91)	0.91 (0.85, 0.96)
Minimal[b]	Ref	0.92 (0.90, 0.94)	0.91 (0.88, 0.94)	0.92 (0.90, 0.95)	Ref	0.85 (0.80, 0.90)	0.89 (0.82, 0.96)	0.93 (0.87, 0.99)
Full[c]	Ref	0.92 (0.90, 0.94)	0.89 (0.87, 0.92)	0.91 (0.88, 0.93)	Ref	0.88 (0.83, 0.93)	0.89 (0.83, 0.96)	0.93 (0.87, 1.00)

[a]Unadjusted, [b]Adjusted for age, diagnosis year, race and ethnicity, nSES, population density, insurance, [c]Adjusted for [b] + stage, receipt of surgery or radiation, Abbreviations: aHR = adjusted hazard ratio, 95%CI = 95% Confidence Interval

© 2025 Korn SM et al. *JAMA Network Open*.
