# Supplementary material for: Travel Time to Treating Facility and Mortality in Men With Prostate Cancer
Source: JAMA Netw Open. 2025 Dec 3;8(12):e2546812. doi: 10.1001/jamanetworkopen.2025.46812 (PMC12676350; doi:10.1001/jamanetworkopen.2025.46812)
Supplement: Supplement 2. — Data Sharing Statement [file jamanetwopen-e2546812-s002.pdf]

Data Sharing Statement

Data

Data available: No

Additional Information

Explanation for why data not available: Data for this study were requested from each registry for the purposes of this research, and Institutional Review Board and Data Use Agreements between Rutgers, Dana-Farber, and each registry prohibit sharing of these data outside of the research team. Inquiries can be directed to: hi97@cinj.rutgers.edu
